# Single-Cell RNA Sequencing Reveals Monocyte-Derived Interstitial Macrophages with a Pro-Fibrotic Phenotype in Bleomycin-Induced Pulmonary Fibrosis

**DOI:** 10.3390/ijms252111669

**Published:** 2024-10-30

**Authors:** Shunli Wang, Jie Li, Caixia Wu, Zhengyao Lei, Tong Wang, Xinxin Huang, Suxia Zhang, Yuting Liu, Xiaohan Bi, Fanshuo Zheng, Xuyou Zhu, Ziling Huang, Xianghua Yi

**Affiliations:** 1 Department of Pathology, Tongji Hospital, School of Medicine, Tongji University, Shanghai 200065, China; 2 Department of Pathology, Fudan University Shanghai Cancer Center, Shanghai 200032, China; 3 Department of Oncology, Shanghai Medical College, Fudan University, Shanghai 200032, China

**Keywords:** single-cell RNA sequencing, idiopathic pulmonary fibrosis, bleomycin, interstitial macrophages, monocyte-derived macrophages, cell–cell communication

## Abstract

Idiopathic pulmonary fibrosis (IPF) is a chronic, progressive lung disease with limited effective therapies. Interstitial macrophages (IMs), especially those derived from monocytes, play an unknown role in IPF pathogenesis. By using single-cell RNA sequencing (scRNA-seq), bleomycin (BLM)-induced pulmonary fibrosis mouse lungs were analyzed to characterize the cellular landscape and heterogeneity of macrophages in this model. scRNA-seq was used to identify distinct interstitial macrophage subpopulations in fibrotic lungs, with monocyte-derived macrophages exhibiting a pro-fibrotic gene expression profile enriched in wound healing, extracellular matrix (ECM) remodeling, and pro-fibrotic cytokine production functions. A pseudotime analysis revealed that IMs originated from monocytes and differentiated along a specific trajectory. A cell–cell communication analysis demonstrated strong interactions between monocyte-derived interstitial macrophages (Mo-IMs) and fibroblasts through the transforming growth factor beta (TGFβ), secreted phosphoprotein 1 (SPP1), and platelet-derived growth factor (PDGF) signaling pathways. Flow cytometry validated the presence and expansion of Mo-IMs subpopulations in BLM-treated mice. This study reveals the cellular heterogeneity and developmental trajectory of lung macrophages in early BLM-induced pulmonary fibrosis, highlighting the crucial role of Mo-IMs with a pro-fibrotic phenotype in IPF pathogenesis via interactions with fibroblasts. Targeting these specific macrophage subpopulations and associated signaling pathways may provide novel therapeutic strategies for IPF.

## 1. Introduction

Idiopathic pulmonary fibrosis (IPF) is a chronic, progressive, and fatal interstitial lung disease characterized by excessive extracellular matrix (ECM) deposition and the architectural distortion of the lung parenchyma, ultimately leading to respiratory failure and death [1,2]. Despite extensive research efforts, the pathogenesis of IPF remains poorly understood, and effective therapies are limited [3,4]. Recent studies have highlighted the role of the innate immune system, particularly macrophages, in the development and progression of IPF [5,6]. Macrophages are highly plastic cells that can be classified into different subpopulations based on their origin, function, and location [7,8]. In the lung, macrophages can be broadly divided into alveolar macrophages (AMs) and interstitial macrophages (IMs) [9,10]. While AMs have been extensively studied in the context of IPF [11,12], the role of IMs, especially those derived from monocytes, remains largely unknown.

Recent advances in scRNA-seq have revolutionized our understanding of cellular heterogeneity and gene expression profiles in complex tissues [13,14]. This powerful technology allows for the identification of rare cell types and the discovery of novel cell subpopulations [15,16]. In the context of IPF, scRNA-seq has been applied to investigate the cellular landscape of human lung fibrosis [17,18] and to characterize the immune cell populations in mouse models of pulmonary fibrosis [19,20]. However, a comprehensive analysis of the heterogeneity and developmental trajectory of lung macrophages, particularly monocyte-derived interstitial macrophages (Mo-IMs), in the early stage of IPF is still lacking.

In this study, we employed scRNA-seq to characterize the cellular landscape and heterogeneity of lung macrophages in a BLM-induced pulmonary fibrosis mouse model. We aimed to identify the subpopulations of Mo-IMs and their potential roles in the early stage of IPF. Our results revealed distinct subpopulations of IMs, with monocyte-derived macrophages exhibiting a pro-fibrotic gene expression profile enriched in functions related to wound healing, extracellular matrix remodeling, and pro-fibrotic cytokine production. A pseudotime analysis demonstrated that IMs originate from monocytes and undergo differentiation along a specific trajectory. Furthermore, a cell–cell communication analysis highlighted strong interactions between Mo-IMs and fibroblasts through the TGFβ, SPP1, and PDGF signaling pathways. Flow cytometry analysis validated the presence and expansion of Mo-IM subpopulations in BLM-treated mice. These findings provide novel insights into the cellular and molecular mechanisms underlying the pathogenesis of IPF and may guide the development of targeted therapies focusing on specific macrophage subpopulations and their associated signaling pathways.

## 2. Results

### 2.1. BLM-Induced Pulmonary Fibrosis in Mice

**Figure 1:**
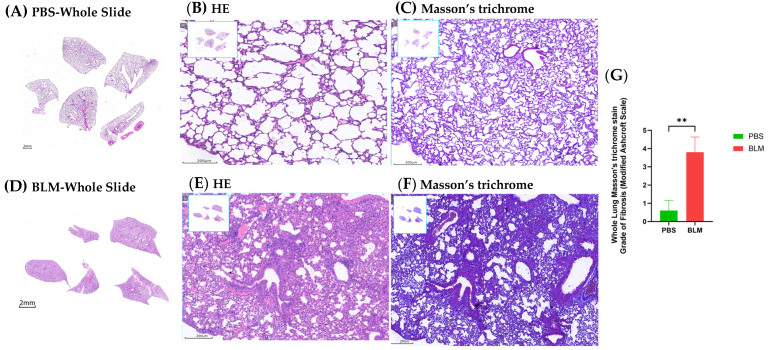
BLM induces pulmonary fibrosis in mice. (**A**–**C**) Representative images of hematoxylin and eosin (HE) and Masson’s trichrome staining from whole lung sections of PBS-treated control mice reveal intact lung architecture with minimal collagen deposition. (**D**–**F**) In contrast, lung sections from the BLM-treated mice display significant epithelial thickening and extensive collagen accumulation, as evidenced by the increased blue staining in the Masson’s trichrome-stained sections, indicating the development of fibrotic lesions. (**G**) The quantification of pulmonary fibrosis using the modified Ashcroft score demonstrates a significant increase in the BLM group (3.80 ± 0.8367) compared to the control group (0.60 per group; Student’s *t*-test: ** *p* < 0.01).

To investigate the effect of BLM on experimental lung fibrosis, wild-type mice were treated with a low concentration of BLM. Pathological analysis via HE and Masson staining (of whole lung tissues from the BLM group on day 7) revealed a significant increase in fibrotic lesions characterized by epithelial thickening and collagen deposition. The modified Ashcroft score, which quantifies the degree of fibrosis, was significantly higher in the BLM group compared to the control group, which is consistent with previous findings [21]. Soluble collagen deposition was markedly increased in BLM-treated mice (Figure 1D–F) compared to PBS-treated controls (Figure 1A–C). The fibrotic scores were significantly higher in the experimental group (3.80 ± 0.8367) compared to the control group (0.60 ± 0.5477, *p* = 0.028 < 0.05), indicating the robust induction of fibrosis by BLM (Figure 1G). 

### 2.2. scRNA-Seq Reveals Distinct Subpopulations of IMs in BLM-Induced Pulmonary Fibrosis

**Figure 2:**
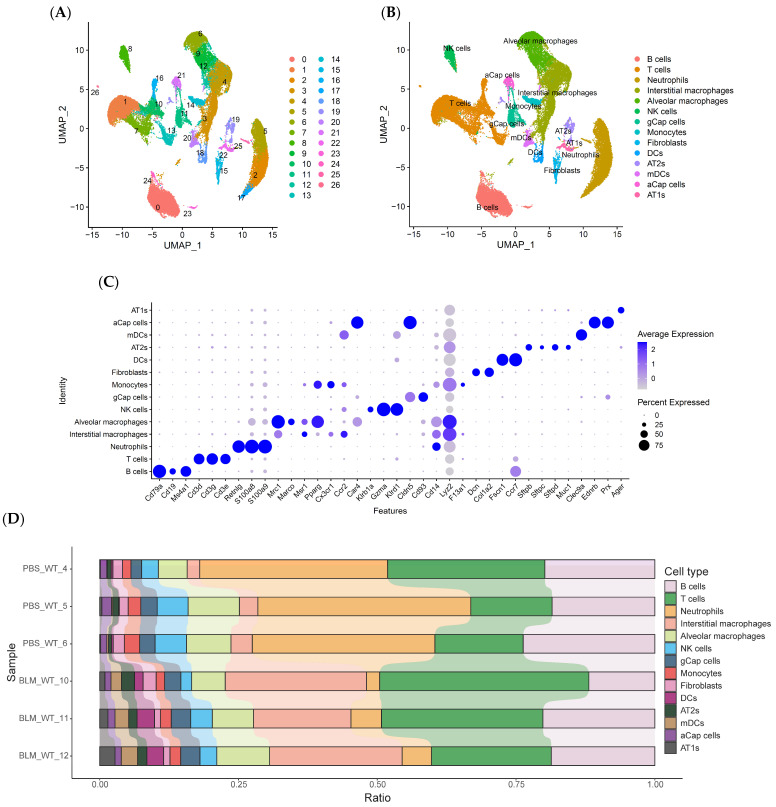
scRNA-seq revealed distinct subpopulations of IMs in BLM-induced pulmonary fibrosis. (**A**) UMAP plot of 54,882 high-quality cells from lung tissues of mice 7 days after BLM or PBS instillation, revealing 27 distinct cell clusters. (**B**) UMAP plots highlighting the distribution of annotated cell types, including alveolar macrophages (AMs), interstitial macrophages (IMs), monocytes, neutrophils, dendritic cells, T cells, B cells, epithelial cells, and fibroblasts, based on the expression of known marker genes. (**C**) Dot plot showing the expression of representative marker genes used for cell type annotation, validating the accuracy of the clustering and cell type identification. (**D**) Comparison of the proportions of IM between PBS and BLM-treated mice, indicating a significant increase in IMs in the BLM group.

To investigate cellular heterogeneity and identify novel cell populations in the early stage of BLM-induced pulmonary fibrosis, we performed scRNA-seq on lung tissues obtained from mice 7 days after BLM instillation. After quality control and data filtering, a total of 54,882 high-quality cells were retained for further analysis. Unsupervised clustering was used to identify 27 distinct cell clusters, which were visualized using uniform manifold approximation and projection (UMAP) (Figure 2A). The cell populations were annotated as alveolar macrophages (Ams), interstitial macrophages (Ims), monocytes, neutrophils, dendritic cells, T cells, B cells, epithelial cells, and fibroblasts based on the expression of known marker genes (Figure 2B,C). Among these clusters, we found that Ims significantly increased in BLM-treated mice compared to control mice (Figure 2D), suggesting their potential role in the pathogenesis of pulmonary fibrosis.

### 2.3. Monocyte-Derived Macrophages Exhibit a Pro-Fibrotic Gene Expression Profile

**Figure 3:**
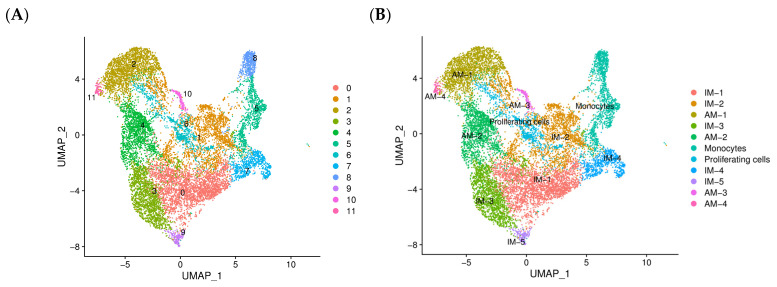

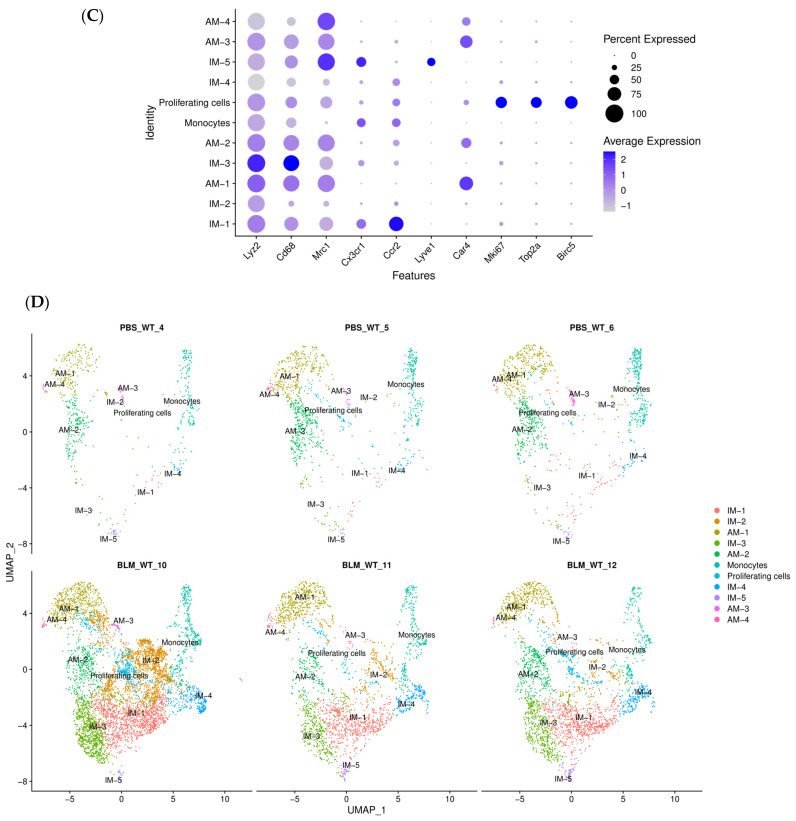

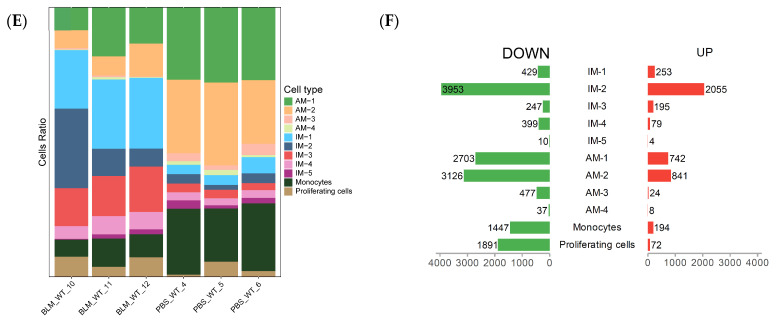

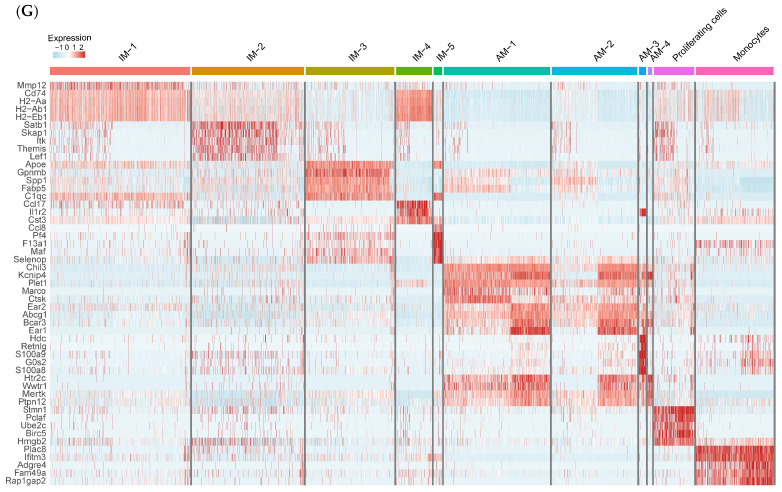

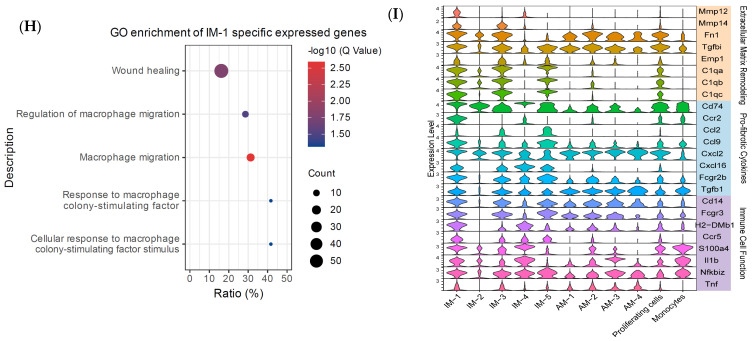
Monocyte-derived macrophages exhibit a pro-fibrotic gene expression profile. (**A**) UMAP plot displaying 12 distinct clusters of monocytes and macrophages identified using the subclustering analysis of 13,214 cells, revealing the heterogeneity of macrophage populations in the fibrotic lung micro-environment. (**B**) UMAP plots highlighting the distribution of each macrophage subtype (AM-1, AM-2, AM-3, AM-4, IM-1, IM-2, IM-3, IM-4, IM-5, and proliferating macrophages) and monocytes, facilitating the visualization of their relative abundances and spatial relationships. (**C**) Dot plot showing the expression of specific markers used to define the 11 macrophage subtypes, validating their distinct identities and functional characteristics. (**D**,**E**) Comparison of the proportions of IM-1, IM-3, IM-4, and IM-5 cells between the PBS and BLM groups, revealing a significant increase in IM-1, IM-3, and IM-4 cells following BLM treatment; this suggests their potential involvement in the fibrotic process. (**F**) Bar plot depicting the number of differentially expressed genes (DEGs) in each macrophage subtype, with a predominance of downregulated genes post-BLM treatment, indicating a shift in gene expression profiles in response to the fibrotic stimulus. (**G**) Heatmap analysis, illustrating highly expressed genes in each macrophage subtype; this highlights the unique transcriptional signatures that define their functional specialization. (**H**) Gene ontology analysis of IM-1-associated genes, indicating enrichment in biological processes related to wound healing, extracellular matrix remodeling, and pro-fibrotic cytokine production, supporting the hypothesis that IM-1 cells play a crucial role in driving the fibrotic response. (**I**) Violin plot displaying the expression of selected genes involved in these pro-fibrotic processes across the macrophage subtypes, further emphasizing the distinct functional profiles of monocyte-derived macrophages in the context of pulmonary fibrosis. These findings provide compelling evidence for the critical role of monocyte-derived macrophages, particularly IM-1 cells, in promoting a pro-fibrotic micro-environment through the expression of genes related to wound healing, matrix remodeling, and cytokine production, thereby contributing to the pathogenesis of IPF.

To further characterize the heterogeneity of macrophages, we performed subclustering analysis on monocytes and macrophages, resulting in the classification of 13,214 cells into 12 distinct clusters (Figure 3A). By utilizing specific markers, we identified 11 different macrophage subtypes: AM-1, AM-2, AM-3, AM-4, IM-1, IM-2, IM-3, IM-4, IM-5, proliferating macrophages, and monocytes (Figure 3B,C). IM-1 to IM-5 exhibited unique gene expression profiles, suggesting distinct functions and developmental trajectories:

IM-1 (Ccr2, H2-EB1, Cx3cr1): Highly expressed genes related to wound healing, extracellular matrix remodeling (e.g., Mmp12, Mmp14), pro-fibrotic cytokines (e.g., Tgfb1, Pdgfb), and immune cell function (e.g., Cd86, H2-Ab1), likely derived from monocytes; they possess a pro-fibrotic phenotype.

IM-2 (Satb1, Skap1): Identified exclusively in the BLM-WT-10 sample and likely composed of low-quality cells; they were excluded from further analysis.

IM-3 (Mrc1, SPP1, Gpnmb): Highly expressed genes related to immune response and antigen presentation. They likely play a role in modulating the immune micro-environment in fibrotic lungs.

IM-4 (CCR2, CCl17): Highly expressed genes related to chemotaxis and cellular migration. They are potentially involved in the recruitment of other immune cells to the fibrotic site.

IM-5 (Mrc1, Cx3cr1, Lyve1): Consistently present in both PBS and BLM treatment groups; they may represent resident interstitial macrophages (Resident-IMs) with homeostatic functions.

The proportions of IM-1, IM-3, and IM-4 cells significantly increased in the BLM group (Figure 3D,E). Furthermore, a differential expression analysis among the subtypes revealed that, following treatment, the number of downregulated genes surpassed that of upregulated genes (Figure 3F). The heatmap analysis highlighted the genes that were highly expressed in each subtype (Figure 3G). Notably, IM-1-associated genes were predominantly enriched in functions related to wound healing (Figure 3H), extracellular matrix remodeling (e.g., Mmp12, Mmp14), pro-fibrotic cytokines (e.g., Tgfb1, Pdgfb), and immune cell function (e.g., Cd86, H2-Ab1) (Figure 3I). These findings suggest that monocyte-derived macrophages exhibit a pro-fibrotic gene expression profile, which may play a crucial role in the pathophysiology of pulmonary fibrosis.

### 2.4. Pseudotime Analysis Reveals That IMs Originate from Monocytes

**Figure 4:**
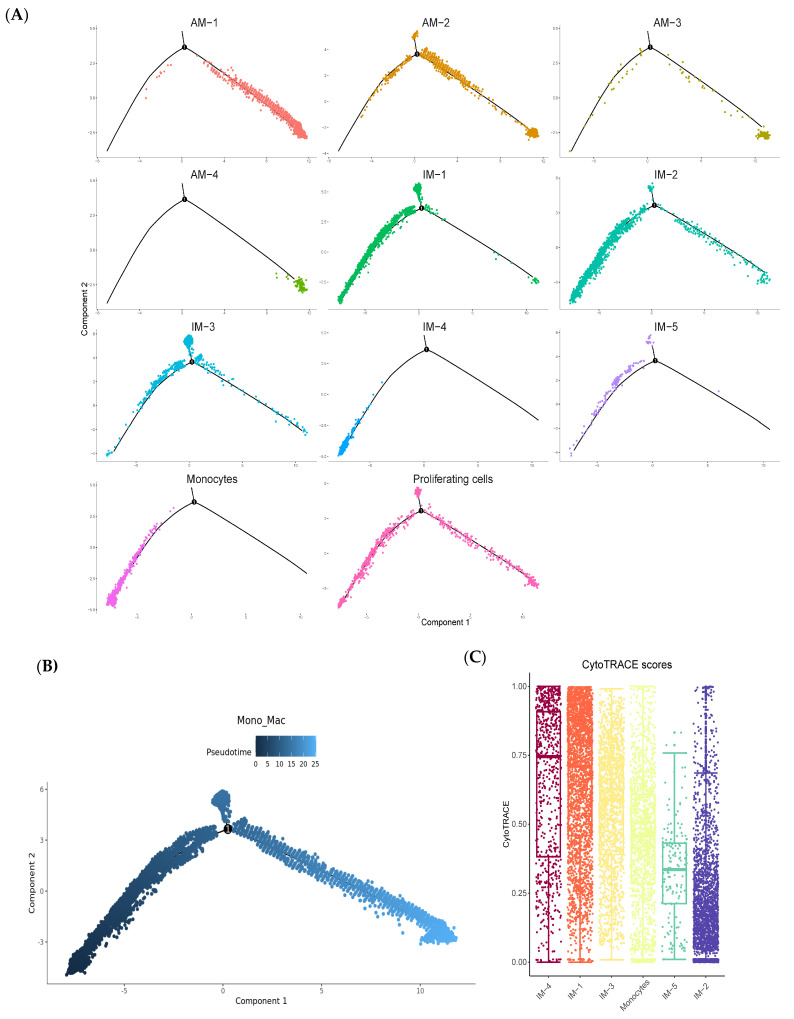

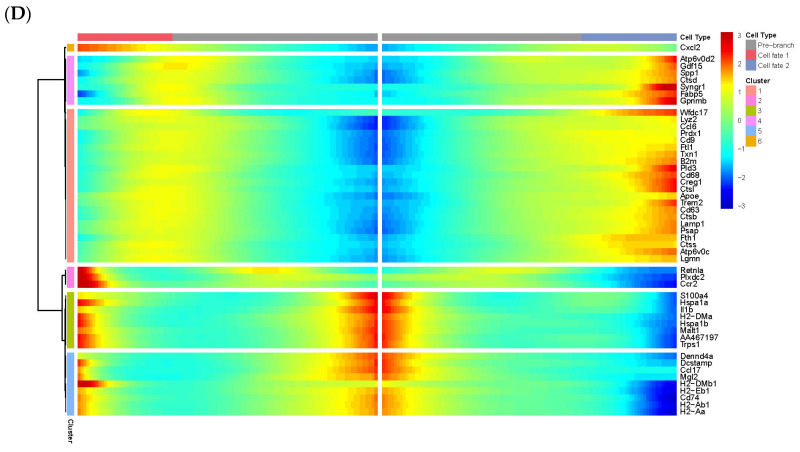
Pseudotime analysis reveals that IMs originate from monocytes. (**A**,**B**) Pseudotime trajectory analysis of AMs, IMs, and monocytes using Monocle 2, displaying a nearly unbranched developmental pathway. The positioning of IM subtypes along the segment associated with monocytes, while the AM subtypes are predominantly located at the opposite end of the trajectory, suggests that IMs primarily originate from monocytes and undergo differentiation along this pathway. (**C**) CytoTRACE analysis of the IM subtypes, revealing a potential differentiation sequence: IM-4, IM-1, IM-3, and IM-5. This analysis provides insights into the developmental relationships among the IM subpopulations and highlights the dynamic nature of macrophage differentiation in response to the fibrotic micro-environment. (**D**) Heatmap depicting gene expression patterns along the pseudotime trajectory, with ‘Per-branch’ corresponding to IM-4, ‘Cell fate1’ encompassing IM-1 and IM-5, and ‘Cell fate2’ corresponding to IM-3. The differential gene expression profiles observed along the trajectory underscore the transcriptional changes that occur during the differentiation process and provide a basis for understanding the functional specialization of each IM subtype.

To investigate the developmental trajectory of lung macrophages in BLM-induced pulmonary fibrosis, we performed a pseudotime analysis on AMs, IMs, and monocytes using the Monocle 2 package. The trajectory analysis revealed that these cell types form a nearly unbranched developmental pathway. Specifically, the IM subtypes were positioned along the segment associated with monocytes, while the AM subtypes were predominantly located at the opposite end of the trajectory (Figure 4A,B). This positioning suggests that the IM subtypes primarily originate from monocytes and undergo differentiation along this pathway.

Further analysis of the IM subtypes using CytoTRACE identified a potential differentiation sequence: IM-4, IM-1, IM-3, and IM-5 (Figure 4C). In the accompanying heatmap, ‘Per-branch’ corresponds to IM-4, ‘Cell fate1’ encompasses IM-1 and IM-5, and ‘Cell fate2’ corresponds to IM-3 (Figure 4D). These findings underscore the monocyte-derived nature of IMs and elucidate the differentiation dynamics within the pulmonary micro-environment in the context of fibrosis.

### 2.5. Cell–Cell Communication Analysis Reveals Strong Interactions Between Mo-IMs and Fibroblasts Through the TGFβ, SPP1, and PDGF Signaling Network

**Figure 5:**
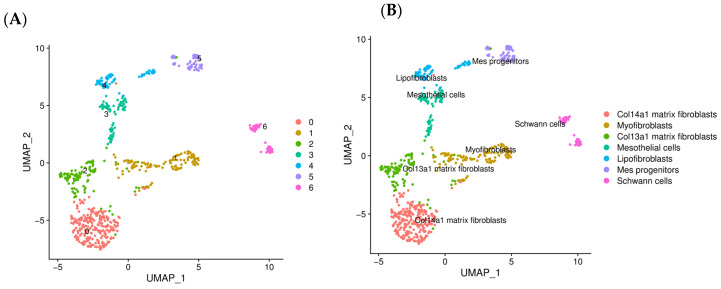

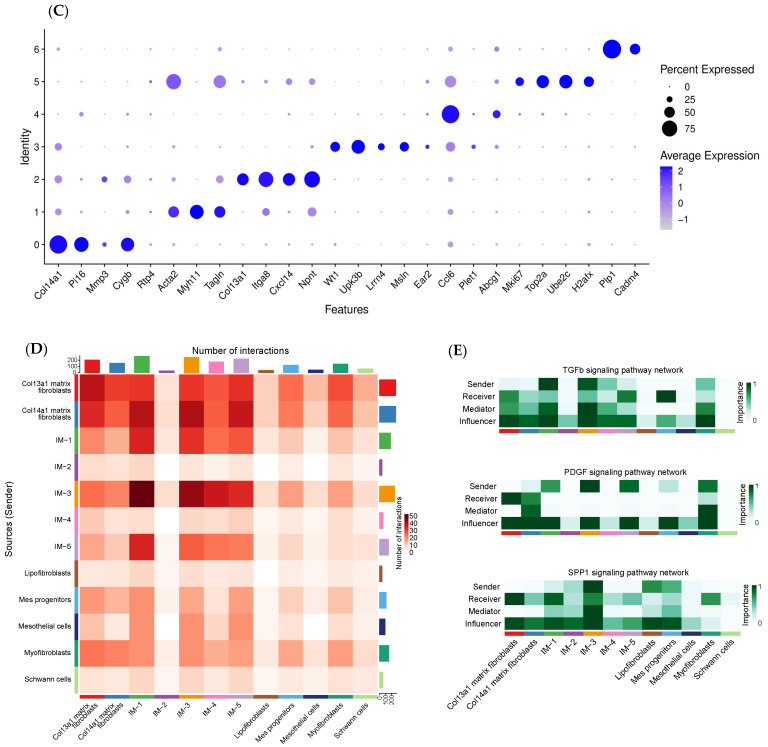

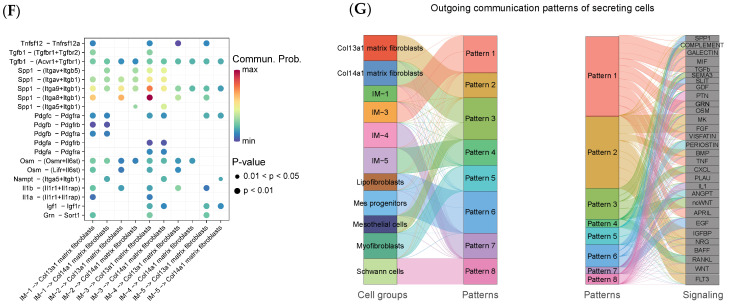
Cell–cell communication analysis reveals strong interactions between monocyte-derived IMs and fibroblasts through the TGFβ, SPP1, and PDGF signaling networks. (**A**) UMAP plot displaying seven distinct clusters of fibroblasts identified using subclustering analysis, revealing the heterogeneity of fibroblast populations in the fibrotic lung micro-environment. (**B**) UMAP plot highlighting the distribution of each fibroblast subtype, including Col14a1 matrix fibroblasts, myofibroblasts, Col13a1 matrix fibroblasts, mesothelial cells, lipofibroblasts, mesenchymal progenitors, and Schwann cells, based on the expression of subtype-specific markers. (**C**) Dot plot showing the expression of representative markers used to define the seven fibroblast subtypes, validating their distinct identities and functional characteristics. (**D**) Heatmap highlighting significant communication events between IMs and fibroblast subtypes, with notable interactions between IM1, IM3, IM5, and fibroblast subtypes expressing Col14a1 matrix fibroblasts, myofibroblasts, and Col13a1 matrix fibroblasts. These interactions suggest a coordinated response between macrophages and fibroblasts in driving the fibrotic process. (**E**) Ligand–receptor interaction diagram illustrating the key signaling pathways involved in the communication between IMs and fibroblasts, with a focus on TGFβ, SPP1, and PDGF signaling networks. (**F**) Signaling pathway network depicting the interactions between IMs and fibroblasts, revealing the central role of TGFβ, SPP1, and PDGF signaling in mediating their cellular communication and potential contribution to fibrosis development. (**G**) “Outgoing communication patterns of secreting cells” analysis, highlighting the interaction between IM1 and TGFβ as the most significant among these pathways; this underscores the critical role of IM1 cells in orchestrating the fibrotic response through the secretion of pro-fibrotic cytokines.

To investigate the heterogeneity of fibroblasts in IPF, we performed a subclustering analysis of fibroblasts, which resulted in the identification of seven distinct clusters (Figure 5A). By using subtype-specific markers, these clusters were further defined into seven different fibroblast subtypes: Col14a1 matrix fibroblasts, myofibroblasts, Col13a1 matrix fibroblasts, mesothelial cells, lipofibroblasts, Mes progenitors, and Schwann cells (Figure 5B,C).

To elucidate the potential interactions between Mo-IMs and fibroblasts in the pathogenesis of IPF, we conducted cell–cell communication analysis using the CellChat package across all fibroblast and IM subtypes. The resulting heatmap highlights significant communication events between these subtypes (Figure 5D). Notably, we observed a significant number of communication events between IM1, IM3, IM5, and fibroblast subtypes expressing Col14a1 matrix fibroblasts, myofibroblasts, and Col13a1 matrix fibroblasts.

To further investigate the key signaling pathways involved in the communication between IMs and fibroblasts, we generated a ligand–receptor interaction diagram (Figure 5E) and a signaling pathway network (Figure 5F). The analysis revealed that the TGFβ, SPP1, and PDGF signaling pathways played the most important roles in the cellular communication between IMs and fibroblasts. Moreover, the “outgoing communication patterns of secreting cells” analysis (Figure 5G) highlighted that the interaction between IM1 and TGFβ was the most significant among these pathways.

### 2.6. Flow Cytometry Analysis Validates the Presence of IM Subpopulations in BLM-Induced Pulmonary Fibrosis

To validate the presence and dynamics of IM subpopulations in the lungs of BLM-treated mice, we performed flow cytometry analysis on cells isolated from enzymatically digested mouse lungs. After pre-gating on FSC-A/SSC-A, excluding doublets, and gating on live cells, immune cells were identified using CD45 staining (Figure S1A–D). Macrophages were further gated as CD64+MerTK+ cells within the CD45+ population (Figure S1E) and classified into CD11b-CD11c+ AM, CD11b+CD11c+ Mo-IMs, and CD11b+CD11c- Resident-IMs (Figure S1F).

Consistent with our scRNA-seq data, we observed a significant increase in both the percentage and absolute number of Mo-IM cells in the lungs of BLM-treated mice at day 7 post-treatment, compared to PBS-treated controls (64.4% vs. 4.09%). In contrast, the percentage and number of Resident-IMs remained unchanged, validating the specific expansion of Mo-IMs in the fibrotic lung environment (Figure S1F).

**Figure 6:**
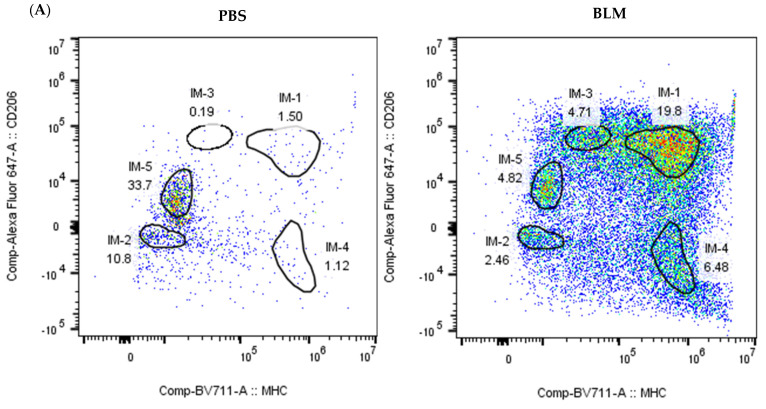

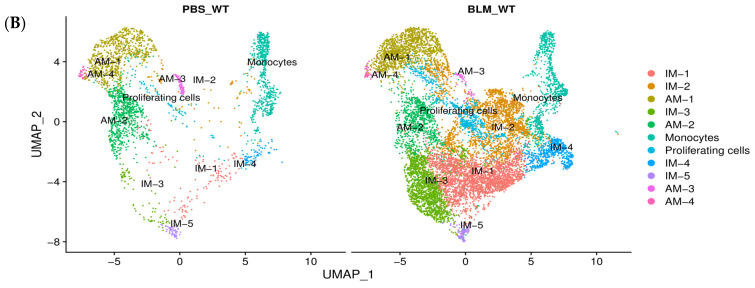

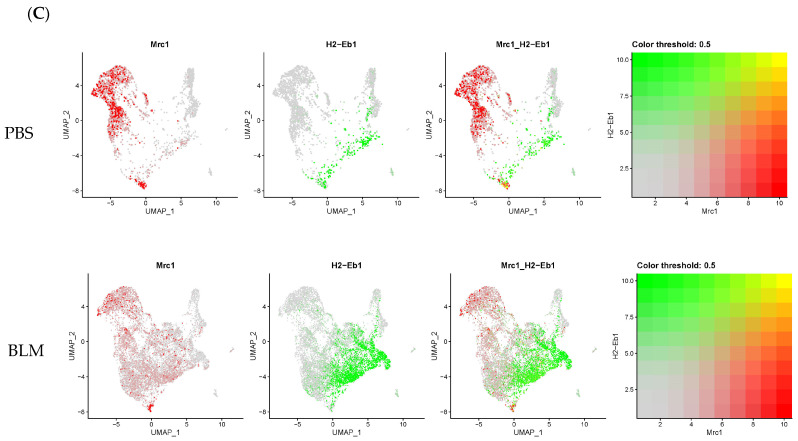
Flow cytometry analysis confirms the presence of distinct IM subpopulations in bleomycin (BLM)-induced pulmonary fibrosis. (**A**) Further analysis of IM subpopulations using surface markers CD206 and MHC class II effectively distinguished five IM subpopulations: IM-1 (MHCHigh+ CD206High+), IM-2 (MHC- CD206-), IM-3 (MHC- CD206High+), IM-4 (MHC+ CD206-), and IM-5 (MHC- CD206Low+). (**B**,**C**) IM1 cells exhibit high expression of both CD206 and MHC class II, which is consistent with the scRNA-seq results, indicating a monocyte-derived pro-fibrotic phenotype.

To further characterize the IM subpopulations (Figure S1G) identified via scRNA-seq, we assessed the expression of surface markers CD206 and MHC class II using flow cytometry. Our analysis revealed that CD206 and MHC class II effectively distinguished five IM subpopulations: IM-1 (MHCHigh+ CD206High+), IM-2 (MHC- CD206-), IM-3 (MHC- CD206High+), IM-4 (MHC+ CD206-), and IM-5 (MHC- CD206Low+) in lung tissues from BLM-treated mice (Figure 6A). Consistent with the scRNA-seq results, IM1 exhibited high expression of both CD206 and MHC class II, indicating the conclusion that these cells are derived from monocytes and possess a pro-fibrotic phenotype (Figure 6B,C).

## 3. Discussion

In this study, we employed scRNA-seq to investigate the cellular heterogeneity and developmental trajectory of lung macrophages in the early stage of BLM-induced pulmonary fibrosis. Our results identified distinct subpopulations of IMs, particularly those derived from monocytes, which exhibited a pro-fibrotic gene expression profile. These findings support our hypothesis that Mo-IMs play a crucial role in the pathogenesis of IPF through their interactions with fibroblasts.

The identification of Mo-IMs with a pro-fibrotic phenotype is consistent with recent studies highlighting the importance of macrophages in the development and progression of IPF. For instance, Aran et al. [11] utilized scRNA-seq to characterize the immune landscape in human lung fibrosis and identified a distinct macrophage subpopulation associated with disease progression. Similarly, Reyfman et al. [12] demonstrated the presence of pro-fibrotic monocyte-derived alveolar macrophages (Mo-AMs) in the lungs of patients with IPF. However, our study provides a more comprehensive understanding of the cellular heterogeneity and developmental trajectory of these macrophages at the single-cell level in a mouse model of pulmonary fibrosis.

The authors performed subclustering analysis on monocytes and macrophages to further characterize their heterogeneity in the context of pulmonary fibrosis. The analysis revealed 12 distinct clusters, including 11 macrophage subtypes and monocytes, each with unique gene expression profiles suggesting distinct functions and developmental trajectories. Among the identified macrophage subtypes, IM-1, IM-3, and IM-4 cells showed significantly increased proportions in the BLM-treated group compared to the PBS control. Notably, IM-1 cells, which likely derive from monocytes, exhibited a pro-fibrotic phenotype with high expression of genes related to wound healing, extracellular matrix remodeling (e.g., Mmp12, Mmp14), pro-fibrotic cytokines (e.g., Tgfb1, Pdgfb), and immune cell function (e.g., Cd86, H2-Ab1). These findings suggest that monocyte-derived macrophages play a crucial role in the pathophysiology of pulmonary fibrosis by promoting a pro-fibrotic micro-environment. The authors also found that IM-3 cells, characterized by the high expression of genes related to immune response and antigen presentation, may contribute to the modulation of the immune micro-environment in fibrotic lungs, whereas IM-4 cells, which have high expression of genes related to chemotaxis and cellular migration, potentially facilitate the recruitment of other immune cells to the fibrotic site.

The pseudotime analysis revealed that IMs originate from monocytes and undergo differentiation along a specific trajectory, which may be influenced by the fibrotic micro-environment in the lung. This finding is consistent with the work of Misharin et al. [22], who demonstrated that Mo-AMs drive lung fibrosis and persist in the lung over the lifespan in a mouse model. Our study extends this knowledge by providing a detailed characterization of the differentiation process and the distinct subpopulations of Mo-IMs in the context of pulmonary fibrosis. The trajectory analysis, performed using the Monocle 2 package on AMs, IMs, and monocytes in a bleomycin BLM-induced pulmonary fibrosis model, revealed a nearly unbranched developmental pathway. CytoTRACE analysis identified a potential differentiation sequence among the IM subtypes: IM-4, IM-1, IM-3, and IM-5. These findings highlight the monocyte-derived nature of IMs and elucidate their differentiation dynamics within the fibrotic pulmonary micro-environment.

The cell–cell communication analysis further emphasized the importance of Mo-IMs in the pathogenesis of IPF by demonstrating their strong interactions with fibroblasts through the TGFβ, SPP1, and PDGF signaling pathways. These findings are consistent with the well-established roles of these signaling pathways in the development of fibrosis. TGFβ is a potent pro-fibrotic cytokine that promotes the activation and differentiation of fibroblasts into myofibroblasts, leading to excessive ECM deposition [23,24]. SPP1 (osteopontin) has been shown to promote fibroblast migration, adhesion, and survival, contributing to the development of pulmonary fibrosis [25]. PDGF is another essential mediator of fibrosis, stimulating fibroblast proliferation and ECM production [26,27]. We conducted cell–cell communication analysis using the CellChat package across all fibroblast and IM subtypes. The resulting heatmap indicated potential communication events among these subtypes, with a considerable number of interactions observed between the IM subtypes (IM1, IM3, and IM5) and fibroblast subtypes expressing Col14a1 matrix fibroblasts, myofibroblasts, and Col13a1 matrix fibroblasts. Further investigation of the key signaling pathways involved in the communication between IMs and fibroblasts revealed that the TGFβ, SPP1, and PDGF signaling pathways played the most important roles in the cellular communication between IMs and fibroblasts. Our study provides novel insights into the cellular sources and targets of these signaling pathways, highlighting the crucial role of Mo-IMs in orchestrating the fibrotic process through their interactions with fibroblasts. Furthermore, we utilized the MethSurv tool to explore the impact of DNA methylation on genes linked to fibrosis and cancer, identifying SSP1 (cg00583003) as exhibiting methylation in lung adenocarcinoma and lung squamous carcinoma. The potential implications of these findings in the context of fibrosis and cancer development were discussed. This additional analysis strengthened the molecular basis of our study and highlighted the potential link between fibrosis and cancer [28,29,30].

While our study offers a comprehensive characterization of Mo-IMs in BLM-induced pulmonary fibrosis, it is essential to acknowledge some limitations. First, our findings are based on a mouse model of pulmonary fibrosis, and the translatability of these results to human IPF remains to be determined. Mouse models, such as the BLM-induced pulmonary fibrosis model, have been widely used to study the pathogenesis of IPF and to test potential therapeutic interventions [31,32]. However, these models do not fully recapitulate the complex pathophysiology of human IPF, which is characterized by a progressive and irreversible decline in lung function [1,33]. Therefore, future studies should conduct relevant research on human samples of IPF to confirm the relevance of monocyte-derived macrophages and their associated signaling pathways in the context of the disease.

Second, our study focuses on the early stage of fibrosis development (day 7 post-BLM instillation), and the dynamics of macrophage populations and their interactions with other cell types may vary at different stages of the disease. The BLM-induced pulmonary fibrosis model is characterized by an initial inflammatory phase followed by a fibrotic phase. As the disease progresses, the fibrotic phase becomes more prominent, characterized by the excessive deposition of ECM proteins, such as collagen, and the formation of fibrotic lesions [32,33]. While our study provides valuable insights into the cellular and molecular events occurring during the early stage of fibrosis development, it is crucial to investigate the temporal changes in macrophage subpopulations and their functional roles throughout disease progression. Future studies should employ longitudinal sampling and scRNA-seq analysis to capture the dynamic nature of macrophage heterogeneity and their interactions with other cell types at different stages of IPF. This approach would allow for the identification of stage-specific macrophage subpopulations and their associated gene expression profiles, providing a more comprehensive understanding of the role of macrophages in the initiation, progression, and resolution of pulmonary fibrosis.

Third, the scRNA-seq data provide a snapshot of the cellular landscape and gene expression profiles, but functional validation of the identified macrophage subpopulations and their roles in fibrosis development is necessary. Our study has identified distinct subpopulations of Mo-IMs with a pro-fibrotic gene expression profile, suggesting their potential involvement in the pathogenesis of IPF. However, to confirm their functional roles, additional experiments, such as cell depletion studies, adoptive transfer experiments, and in vitro co-culture assays, should be performed [34,35]. These functional validation experiments will provide a more definitive understanding of the causal relationships between specific macrophage subpopulations and fibrosis development.

Despite these limitations, our study provides a solid foundation for future research on the role of Mo-IMs in the pathogenesis of IPF. Further studies should focus on validating these findings in human IPF samples and exploring the therapeutic potential of targeting specific macrophage subpopulations and their associated signaling pathways. For instance, the selective inhibition of monocyte recruitment to the lung or the modulation of macrophage polarization towards an anti-fibrotic phenotype could be potential strategies to attenuate fibrosis progression [36,37]. Additionally, investigating the interplay between Mo-IMs and other key cell types involved in fibrosis, such as epithelial cells and endothelial cells, could provide a more comprehensive understanding of the complex cellular networks driving IPF pathogenesis [38,39].

## 4. Materials and Methods

### 4.1. Mice

Eight-week-old C57BL/6 mice, procured from Shanghai SLAC Laboratory Animal Co., Ltd. (Shanghai, China), were housed in a specific pathogen-free facility. The mice were treated with Bleomycin (BLM) (Item No. 13877, Cayman Chemical Company, Ann Arbor, MI, USA) to induce lung fibrosis, as previously described [40]. Briefly, the mice were anesthetized and intratracheally instilled with BLM (Dose: 1 μg/μL/g body weight) in a volume of 50 μL PBS. The control mice received PBS alone. Lung tissues were harvested on day 7 after the bleomycin treatment for further analysis. All animal experiments were approved by the Institutional Animal Care and Use Committee of Tongji University (Approval No. TJ-HB-LAC-2023-49).

### 4.2. Histology and Masson’s Trichrome Staining

Hematoxylin and eosin staining were performed using a Leica ST5010 Autostainer XL (Leica Biosystems, Wetzlar, Germany) to ensure the consistent and high-quality staining of tissue sections, following the manufacturer’s instructions. For Masson’s trichrome staining, the slides were processed using a BASO Masson kit (BA4079A, Zhuhai Baso Biotechnology Co., Ltd., Zhuhai, China) according to the manufacturer’s instructions. This staining method was employed to differentiate between collagen and other tissue components. The fibrotic score was quantified following the modified Ashcroft scoring system, as described by Hubner et al. [21]. Whole slide images were scanned at 40× magnification using a Pannoramic MIDI II scanner (3DHISTECH Ltd., Budapest, Hungary). For each lobe, an average score was calculated based on 4 to 15 randomly captured images at 20× magnification, with the number of images varying according to the size of the lobe. Three pathologists, who were blinded to the experimental conditions, independently evaluated the slides. The average score for each mouse was determined based on these evaluations to ensure an unbiased and accurate assessment of fibrosis.

### 4.3. Single-Cell RNA Sequencing Library Construction and Sequencing

scRNA-seq libraries were constructed using the 10x Chromium Single Cell 3′ platform (10X Genomics, Pleasanton, CA, USA) according to the manufacturer’s protocol. Briefly, single-cell suspensions were loaded onto a Chromium microfluidic chip to generate single-cell gel beads in emulsion (GEMs). The scRNA-seq libraries were then prepared using the Chromium Single Cell 3′ Reagent Kit v3.1 (10X Genomics, Pleasanton, CA, USA). Library quality was assessed using the Agilent 2100 Bioanalyzer system (Agilent Technologies, Santa Clara, CA, USA), and sequencing was performed on the Illumina NovaSeq 6000 platform (Illumina, San Di-ego, CA, USA) with a sequencing depth of at least 50,000 reads per cell. The raw sequencing data were deposited in the NCBI Sequence Read Archive (SRA) under accession number PRJNA1162793.

### 4.4. Single-Cell RNA Sequencing Data Analysis

The sequencing data for each sample were aligned to the mouse reference genome mm10-2020-A using CellRanger v7.1.0 (https://www.10xgenomics.com/support/software/cell-ranger, accessed on 1 October 2024) to facilitate cell identification and expression matrix construction. The expression matrix underwent quality control using Seurat v4 (https://satijalab.org/seurat, accessed on 1 October 2024) with the following criteria: (1) cells with an expression of 200 to 5500 genes were retained; (2) cells with less than 15% mitochondrial gene expression were retained; and (3) only genes detected in at least three cells were included. Doublets (instances where two or more cells are contained within a single droplet) were removed using DoubletFinder v2.0.2 (https://github.com/chris-mcginnis-ucsf/DoubletFinder, accessed on 1 October 2024).

Data integration across multiple samples was performed using the canonical correlation analysis (CCA) algorithm within Seurat. The integrated dataset was scaled using the ScaleData function. Principal component analysis (PCA) was conducted on the top 2000 highly variable genes to reduce dimensionality. The top 50 principal components (PCs) were selected using the FindNeighbors function, followed by cell cluster identification through the FindClusters function with a resolution of 0.6. Cell clusters were visualized and further explored using Uniform Manifold Approximation and Projection (UMAP). Marker genes enriched in each cluster were identified using the FindAllMarkers function in Seurat, with a minimum percentage threshold of 0.25. Gene ontology enrichment analysis was performed using the clusterProfiler v3.14.0 package (http://bioconductor.org/packages/clusterProfiler, accessed on 1 October 2024), with parameters set for pvalueCutoff, qvalueCutoff, and pAdjustMethod at 1, 1, and BH, respectively.

### 4.5. Single-Cell Pseudotime Analysis

A pseudotime analysis was conducted using Monocle 2 v2.28.0 (http://cole-trapnell-lab.github.io/monocle-release, accessed on 1 October 2024). The starting point for the pseudotime trajectory was determined using CytoTRACE v0.3.3 (https://cytotrace.stanford.edu, accessed on 1 October 2024). Genes exhibiting high dispersion across cells were selected to arrange the cells of interest. The DDRTree method was employed to partition the data into two components, and the BEAM algorithm was utilized to identify genes associated with branching events.

### 4.6. Cell–Cell Interaction Analysis

Cell–cell communication was inferred and visualized based on known ligand–receptor pair expression using CellChat v2.1.0 (http://www.cellchat.org, accessed on 1 October 2024). All classes of ligand–receptor interactions present in the database were included in the analysis. The netVisualbubble function within CellChat was employed to visualize the differential communication probabilities of bidirectional ligand–receptor pairs.

### 4.7. Flow Cytometry

Single-cell suspensions were prepared from lung tissues, as previously described [41,42]. For staining, the following antibodies were utilized: Ghost Dye™ Red 780 (Cat# 13-0865-T100) (Cytek Biosciences, Fremont, CA, USA), PE/Dazzle™ 594 anti-mouse CD45 (Clone 30-F11, Cat# 103145) (BioLegend, San Diego, CA, USA), Brilliant Violet 421™ anti-mouse MERTK (Clone 2B10C42, Cat# 151510) (BioLegend, San Diego, CA, USA), PE anti-mouse CD64 (Clone X54-5/7.1, Cat# 139303) (BioLegend, San Diego, CA, USA), Brilliant Violet 711™ anti-mouse I-A/I-E (Clone M5/114.15.2, Cat# 107643) (BioLegend, San Diego, CA, USA), Alexa Fluor^®^ 488 anti-mouse CD11c (Clone N418, Cat# 117313) (BioLegend, San Diego, CA, USA), PE/Cyanine7 anti-mouse CD192 (Clone SA203G11, Cat# 150611) (BioLegend, San Diego, CA, USA), PerCP/Cyanine5.5 anti-mouse CD170 (Clone E50-2440, Cat# 155525) (BioLegend, San Diego, CA, USA), Brilliant Violet 785™ anti-mouse/human CD11b (Clone M1/70, Cat# 101243) (BioLegend, San Diego, CA, USA), Alexa Fluor^®^ 647 Rat Anti-Mouse CD206 (Clone MR6F3) (BD Biosciences, San Jose, CA, USA), and Thermo Fisher Scientific FIX & PERM™ Cell Permeabilization Kit (Cat# GAS003) (Thermo Fisher Scientific, Waltham, MA, USA). Data acquisition was conducted using a Cytek Aurora flow cytometer (Cytek Biosciences, Fremont, CA, USA), and analysis was performed using FlowJo 10.8.1 (FlowJo, LLC, a subsidiary of BD Biosciences, Ashland, OR, USA).

### 4.8. Statistical Analysis

Statistical analyses were performed using GraphPad Prism 8.0 software (GraphPad Software, La Jolla, CA, USA). The data are presented as mean ± standard error of the mean (SEM). Comparisons between two groups were made using the unpaired Student’s *t*-test. Multiple group comparisons were performed using a one-way analysis of variance (ANOVA) followed by Tukey’s post hoc test. A *p*-value of <0.05 was considered statistically significant.

## 5. Conclusions

This study revealed the cellular heterogeneity and developmental trajectory of lung macrophages in the early stage of BLM-induced pulmonary fibrosis, highlighting the crucial role of Mo-IMs with a pro-fibrotic phenotype in the pathogenesis of IPF. The strong interactions between these macrophages and fibroblasts through the TGFβ, SPP1, and PDGF signaling pathways provide novel insights into the cellular and molecular mechanisms underlying fibrosis development. These findings open new avenues for research and may guide the development of targeted therapies for IPF by focusing on specific macrophage subpopulations and their associated signaling pathways. Future studies should aim to validate these findings in human IPF samples, investigate the temporal dynamics of macrophage heterogeneity, and functionally characterize the identified macrophage subpopulations to develop more effective and personalized treatment strategies for this devastating disease.

## Data Availability

The raw sequencing data that support the findings of the study have been deposited in the NCBI Sequence Read Archive (SRA) under accession number PRJNA1162793. For reviewer access, the data can be viewed directly via the following link: https://dataview.ncbi.nlm.nih.gov/object/PRJNA1162793?reviewer=emsa5n43q48sllcns2fqk2vpti, accessed on 1 October 2026. All data needed to evaluate the conclusions in the paper are present in the paper or the Supplementary Materials.

## Supplementary Materials

The following supporting information can be downloaded at: https://www.mdpi.com/article/10.3390/ijms252111669/s1.
