# Supplementary material for: Single-Cell RNA Sequencing Reveals Monocyte-Derived Interstitial Macrophages with a Pro-Fibrotic Phenotype in Bleomycin-Induced Pulmonary Fibrosis
Source: Int J Mol Sci. 2024 Oct 30;25(21):11669. doi: 10.3390/ijms252111669 (PMC11545836; doi:10.3390/ijms252111669)
Supplement: Supplementary file 1 [file ijms-25-11669-s001.zip › ijms-3256019-supplementary.pdf]

International Journal of
Molecular Sciences

MDPI

Supplementary Materials for

Single-Cell RNA Sequencing Reveals Monocyte-Derived Interstitial Macrophages with a Pro-Fibrotic Phenotype in Bleomycin-Induced Pulmonary Fibrosis

Shunli Wang et al.

Supplemental Materials

Figure S1: Gating Scheme for Flow Cytometry Analysis Confirms Distinct Interstitial Macrophage Subpopulations in Bleomycin-Induced Pulmonary Fibrosis

(A–D) The initial pre-gating strategy for flow cytometry analysis of lung immune cells is outlined, incorporating several critical steps:

- FSC-A/SSC-A Gating: This step facilitates the identification of cell populations based on their size (FSC-A) and granularity (SSC-A).

- Exclusion of Doublets: This ensures that only single cells are analyzed, enhancing the accuracy of the data.

- Live Cell Gating: The use of a viability dye allows for the exclusion of dead cells from the analysis.

- CD45 Staining: This staining identifies immune cells, ensuring that only relevant populations are considered in subsequent analyses.

This comprehensive gating strategy is essential for the precise identification and characterization of immune cell populations within the complex lung microenvironment.

(E) Macrophages were specifically identified within the CD45+ population as CD64+MerTK+ cells, allowing for the targeted analysis of different macrophage subsets.

(F) Further categorization of macrophages revealed three distinct subsets:

- CD11b-CD11c+ Alveolar Macrophages (AM)

- CD11b+CD11c+ Monocyte-Derived Interstitial Macrophages (Mo-IMs)

- CD11b+CD11c- Resident Interstitial Macrophages (Resident-IMs)

The flow cytometry results indicate a significant increase in both the percentage and absolute number of Mo-IM cells in the lungs of BLM-treated mice compared to control groups (64.4% vs. 4.09%).. This finding corroborates previous single-cell RNA sequencing (scRNA-seq) results, confirming the specific expansion of this macrophage subpopulation within the fibrotic lung environment.

(G) Gating on interstitial macrophage populations, including both CD11b+CD11c+ Mo-IMs and CD11b+CD11c- Resident-IMs, revealed distinct subpopulations within the interstitial macrophage compartment in lung tissues from BLM-treated mice. This highlights the heterogeneity of macrophage responses in the context of pulmonary fibrosis.

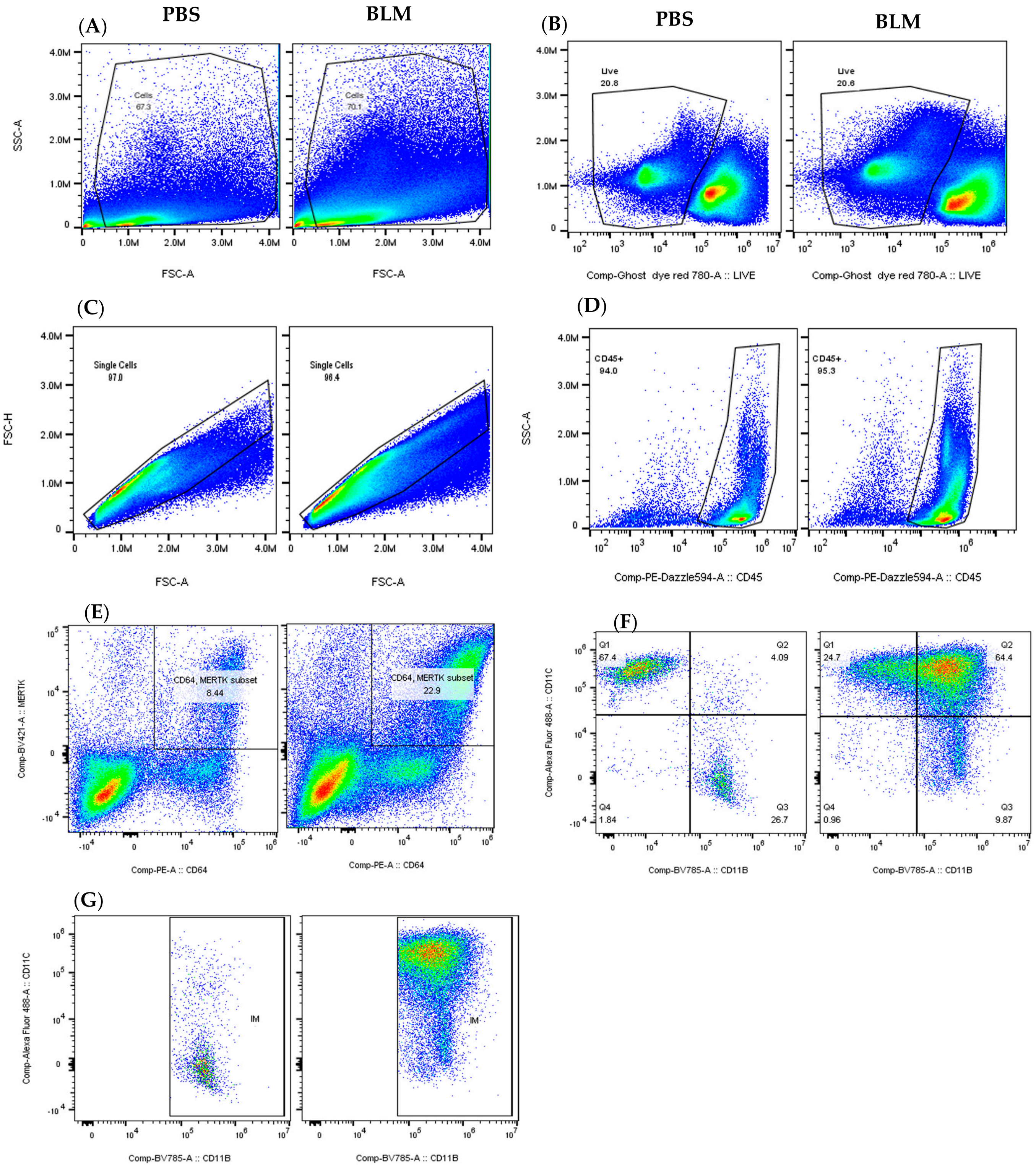

Figure S1. Gating Scheme for Flow cytometry analysis confirms the presence of distinct interstitial macrophage (IM) subpopulations in bleomycin (BLM)-induced pulmonary fibrosis. (**A**–**D**) Basic pre-gating strategy for flow cytometry analysis of lung immune cells, including FSC-A/SSC-A gating to identify cell populations based on size and granularity, the exclusion of doublets to ensure the analysis of single cells, the gating on live cells using a viability dye, and the identification of immune cells via CD45 staining. This rigorous gating strategy ensures the accurate identification and characterization of immune cell populations within the complex lung micro-environment. (**E**) Macrophages were gated as CD64+MerTK+ cells within the CD45+ population, allowing for the specific identification of macrophage subsets. (**F**) Further classification of macrophages into CD11b-CD11c+ alveolar macrophages (AM), CD11b+CD11c+ monocyte-derived interstitial macrophages (Mo-IMs), and CD11b+CD11c- resident interstitial macrophages (Resident-IMs). The flow cytometry data demonstrate a significant increase in both the percentage and absolute number of Mo-IM cells in the lungs of BLM-treated mice compared to controls, validating the scRNA-seq findings and confirming the specific expansion of this macrophage subpopulation in the fibrotic lung environment. (**G**) Gating on IM populations, including both CD11b+CD11c+ Mo-IMs and CD11b+CD11c- Resident-IMs, revealed distinct subpopulations within the interstitial macrophage compartment in lung tissues from BLM-treated mice.
